# Sustainable Doping via Molecular Adsorption on Thin-Film Semiconductor Bi_2_O_2_Se

**DOI:** 10.1021/acs.nanolett.5c04613

**Published:** 2025-12-03

**Authors:** Tai-Ting Lee, Chi-Chun Cheng, Tzu-Wen Kuo, Shu-Yu Hsu, Chun-Yen Hsiao, Yu-Lun Chueh, Po-Wen Chiu, Mei-Yin Chou

**Affiliations:** † Department of Physics, National Taiwan University, Taipei 10617, Taiwan; ‡ Institute of Atomic and Molecular Sciences, 38017 Academia Sinica, Taipei 10617, Taiwan; ¶ Department of Electrical Engineering, 34881 National Tsing Hua University, Hsinchu 30013, Taiwan; § Department of Materials Science and Engineering, 34881 National Tsing Hua University, Hsinchu 30013, Taiwan; ∥ College of Semiconductor Research, 34881 National Tsing Hua University, Hsinchu 30013, Taiwan

**Keywords:** molecular doping, chemisorption, thin-film semiconductor, threshold voltage modulation

## Abstract

Doping in semiconductors is commonly achieved by incorporating foreign atoms into the crystal, which is a complex process that requires precise control. In two-dimensional or thin-film semiconductors, an alternative approach involving surface modification, such as molecular adsorption, has been proposed and attempted. However, successful doping via gas adsorption has not yet been reported. In this work, we present both first-principles calculations and experimental evidence demonstrating the feasibility of this approach in thin-film semiconductor Bi_2_O_2_Se, which has recently gained attention for its high carrier mobility, moderate band gap, and excellent air stability. We find that p-type doping can be achieved through surface adsorption of molecules such as NO_2_, which exhibits stable chemisorption and significant charge transfer. This adsorption-induced p-doping effect significantly modulates the threshold voltage in as-grown n-type samples and remains stable for more than 10 days under ambient conditions, markedly improving electrostatic control and switching behavior in Bi_2_O_2_Se-based devices.

## Graphical abstract

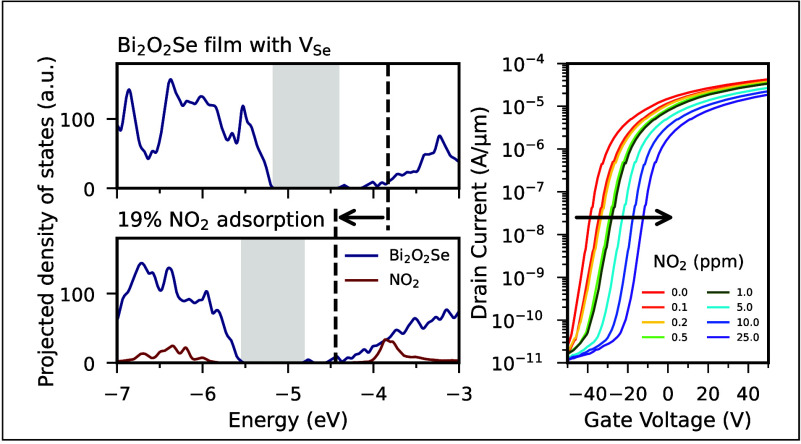

A thin-film semiconductor, Bi_2_O_2_Se, has recently emerged as a highly promising candidate owing to its ultrahigh mobility (470,000 cm^2^/V·s at 2 K and 812 cm^2^/V·s at room temperature^1^), moderate band gap, and good air stability.^2^ These attributes make it suitable for various applications in logic,^2,3^ optoelectronic,^4–6^ thermoelectric,^7,8^ and ferroelectric^9–11^ devices. However, during high-temperature chemical vapor deposition (CVD), selenium vacancies (V_Se_) inherently form in the crystal. These donor-like vacancies give rise to pronounced n-type conductivity and a high carrier concentration (10^19^–10^20^ cm^–3^).^12,13^ As a result, as-grown Bi_2_O_2_Se is an n-type degenerate semiconductor with limited electrostatic gate control in devices. Reliable transistor switching between on- and off-states is essential to preventing functional inefficiencies and excessive power consumption. The high threshold voltage in as-grown Bi_2_O_2_Se samples poses a major barrier to practical device integration.

Previous studies have shown that the carrier concentration in Bi_2_O_2_Se is strongly influenced by the thickness of the film and the concentration of selenium vacancies.^12–14^ Although thinner films improve gate control in devices, they still require large gate voltages to reach the off-state. For example, Wu et al.^2^ reported a threshold voltage of −7 V for a 6.2 nm Bi_2_O_2_Se device with HfO_2_ as the gate oxide. Subsequently, they demonstrated that the concentration of selenium vacancies in Bi_2_O_2_Se can be reduced by using pure selenium powder as a precursor, leading to a lower carrier concentration.^13^ However, a more direct and flexible strategy for carrier modulation in Bi_2_O_2_Se is still needed to realize its full technological potential.

The development of next-generation nanoelectronics requires semiconductors that not only exhibit outstanding intrinsic transport properties but also permit precise and scalable doping control. Precise and stable doping through the incorporation of foreign atoms remains a challenge in thin-film and low-dimensional materials. Because of the reduced dimensionality, surface modification, such as the adsorption of molecules, becomes an alternative and possible doping path, which is a unique and feasible feature in two-dimensional (2D) devices. This may significantly affect channel transport and overall device performance, as demonstrated in doping 2D transition-metal dichalcogenides (TMDs) by organic molecules or ions using solution-based methods,^15–17^ yet stable doping performed in the gas phase has been a challenging task. Gas adsorption on layered transition-metal dichalcogenides (TMDs) has been extensively studied for sensing applications.^18–21^ However, the doping effect in TMDs is often reversible and unsustainable since the adsorbates readily desorb once the external gas is removed.

In contrast, Xu et al.^22^ reported a persistent increase in resistivity following NO_2_ exposure in Bi_2_O_2_Se-based sensors, indicating the retention of molecules even after gas removal and a surface chemistry different from that of TMDs. This can be attributed to the specific surface configuration of Bi_2_O_2_Se that has a composition of charged layers. To balance the charge, the surface termination of Bi_2_O_2_Se in the upper and lower Se planes is characterized by 50% selenium vacancies on the surfaces.^23^ These vacancies can possibly anchor adsorbates and allow for long-lasting adsorption and charge transfer.

In this work, we combine theoretical and experimental approaches to investigate the doping effect through molecular adsorption and show that it is a nondestructive and scalable method of tuning the carrier concentration in the thin-film semiconductor Bi_2_O_2_Se. Using first-principles calculations within density functional theory (DFT), we examine the adsorption of selected molecules on the surfaces of both pristine and defective (with internal Se vacancies) Bi_2_O_2_Se films, analyzing charge transfer, adsorption energetics, and changes in electronic structure. Our results indicate that NO_2_ is a particularly effective and stable p-type surface dopant, promoting the transfer of holes to the underlying channel layers. We also present our experimental measurements demonstrating that the adsorption of NO_2_ allows tunable modulation of the threshold voltage in Bi_2_O_2_Se FETs and effectively enhances transistor control. The induced doping effect remains stable for more than 10 days under ambient conditions. This work establishes a practical approach to overcoming the intrinsic doping bottleneck and switching limitations in Bi_2_O_2_Se. It also demonstrates that molecular adsorption is a practical and sustainable route to doping and provides a broader framework for engineering electronic properties in low-dimensional semiconductors.

First, we performed first-principles calculations within density functional theory (DFT) to investigate changes in the electronic properties upon the adsorption of various molecules. The Bi_2_O_2_Se surface was modeled using a slab with a thickness of three chemical units and terminated with Se layers on both sides with approximately 50% Se vacancies on the surfaces and zero net charge polarization. We used a paired-vacancy model modified from previous theoretical studies,^24^ following the finding by scanning tunneling microscopy (STM).^23^ The computational details and choice of surface vacancy configurations are provided in the Supporting Information. We considered various adsorption configurations using a 3 × 3 or 4 × 4 surface unit cell. The molecular adsorption energy is defined as the absolute value of the total energy difference between the adsorbed system and its isolated components; higher values indicate stronger binding. Selenium vacancies (V_Se_) are commonly observed in CVD-grown Bi_2_O_2_Se films and known to induce n-type degenerate conductivity.^12,13^ To simulate this defect system in our calculation, two Se atoms per 4 × 4 supercell were removed from the inner layers of the slab, producing a defective film with a V_Se_ concentration of 4.1%.

Several representative molecules were considered in our calculation, and their energy levels are shown in Figure 1. The first group includes molecules with positive electron affinity (values shown in parentheses) that are likely to induce charge transfer upon adsorption: NO_2_ (2.27 eV),^25^ NO (0.03 eV),^26^ SO_2_ (1.11 eV),^27^ and Cl_2_ (2.35 eV).^28^ The second group, NH_3_, NF_3_, CO_2_, and N_2_, contains electronegative atoms but exhibits negative electron affinity. To examine possible charge transfer, we compare the energy levels of each molecule with the density of states (DOS) of the pristine and defective (4.1% V_Se_) Bi_2_O_2_Se films in Figure 1, with the common energy zero set at the vacuum level. The molecular HOMO is marked with an asterisk (*), the partially occupied state is indicated by a half-filled circle (◐), and the peak height reflects orbital degeneracy. NO_2_ and NO exhibit spin-polarized states, while the others are spin-degenerate.

**Figure 1:**
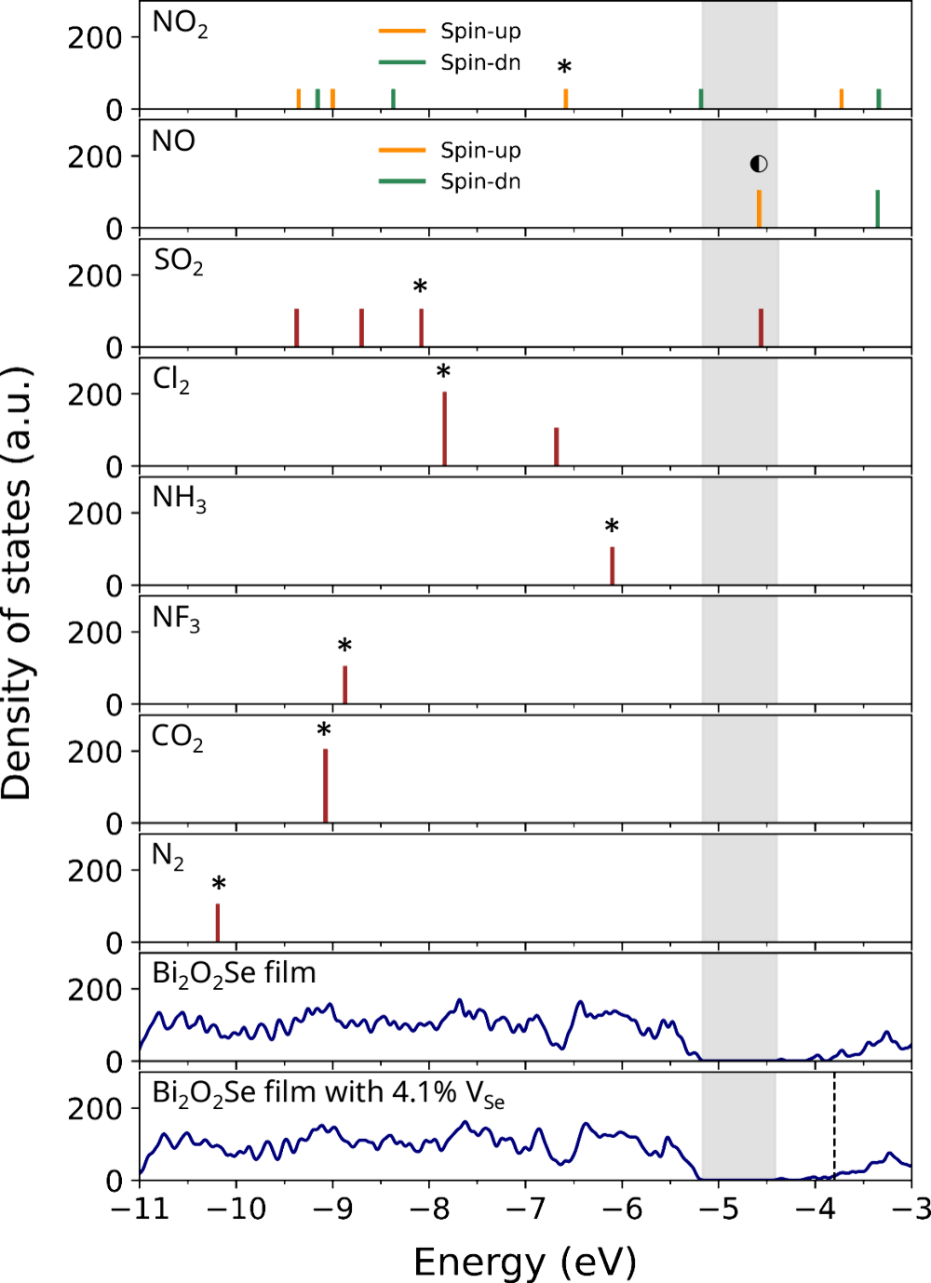
Energy levels of selected molecules compared with the density of states of films of pristine Bi_2_O_2_Se and defective Bi_2_O_2_Se with 4.1% V_Se_. The first group of molecules (NO_2_, SO_2_, NO, Cl_2_) has positive electron affinity, while the second group (NH_3_, NF_3_, CO_2_, N_2_) has negative electron affinity. The HOMO is marked with an asterisk (*), and the partially occupied state is indicated by a half-filled circle (◐). Peak heights reflect orbital degeneracy. The band gap of Bi_2_O_2_Se is highlighted in gray, and the Fermi level of the film with 4.1% internal Se vacancies is marked by a dashed line. All energy levels are with respect to the vacuum level.

In Figure 1 it can be seen that the energies of HOMOs in NH_3_, NF_3_, CO_2_, and N_2_ are well within the valence bands of Bi_2_O_2_Se and that their LUMOs are quite high in energy (outside the energy range of Figure 1), indicating that little interaction with the Bi_2_O_2_Se surface is expected. In contrast, the frontier orbitals of NO_2_, NO, SO_2_, and Cl_2_ are within the energy range of Figure 1. In particular, the LUMOs that will accommodate additional charges in NO_2_, NO, and SO_2_ are close to or within the energy gap of Bi_2_O_2_Se and below the Fermi level (dashed line) of the Bi_2_O_2_Se film with Se vacancies. Therefore, the adsorption of these molecules may give rise to an interesting p-doping effect.

We first studied the adsorption of a single NO_2_ molecule in a 3 × 3 supercell, corresponding to a surface coverage of 11.1% (approximately 0.7 molecule/nm^2^). Figure 2(a) shows the geometry of the pristine film with Se vacancies on the surface. After exploring multiple initial configurations, we found that the adsorbed NO_2_ molecule prefers a Se vacancy site on the surface. This is consistent with previous results of experimental^22,29^ and theoretical^30^ studies that identified Se surface vacancy sites as generally active adsorption centers. Two possible low-energy orientations of the NO_2_ molecule were found, as shown in Figures 2(b) and 2(c). Configuration I [Figure 2(b)] has an adsorption energy of 1.05 eV, with the N atom occupying a Se vacancy site and the two O atoms at a distance of approximately 2.62 Å from the adjacent Bi atoms. Configuration II [Figure 2(c)] has an adsorption energy of 1.14 eV, with an O atom falling into the vacancy site and the other O atom and the N atom remaining elevated above the Se plane. The distance between O (in NO_2_) and Bi (in Bi_2_O_2_Se) is approximately 2.85–3.06 Å. The interaction between NO_2_ and the Bi_2_O_2_Se film is illustrated by the difference charge density plots in Figures 2(b) and 2(c). As determined by Bader charge analysis, the molecule gains 0.80 and 0.79 electron in the two configurations, respectively. The energy difference between these two configurations is small, and the charge transfer values are almost identical. Similar results were found for NO_2_ adsorbed at different Se vacancy sites (see Figure S3 in the Supporting Information).

**Figure 2:**
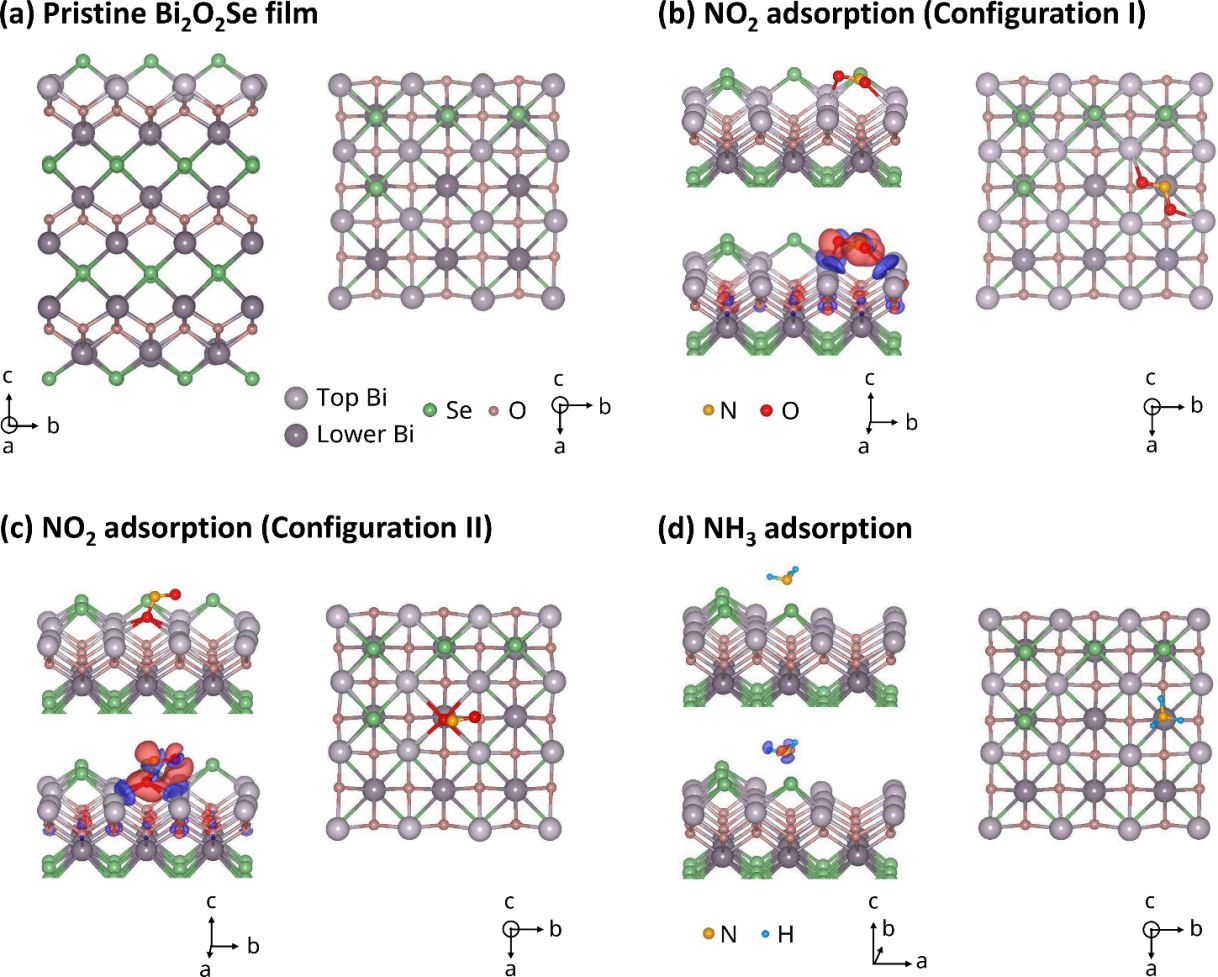
Optimized geometric structures and difference charge density plots. (a) Pristine Bi_2_O_2_Se film (side view left; top view right) with 50% Se vacancies in the Se plane on the surfaces; (b) NO_2_ adsorption in the first configuration; (c) NO_2_ adsorption in the second configuration; (d) NH_3_ adsorption. Shown in (b)–(d) are a side view (left) with the corresponding difference charge density plot and a top view (right). Charge accumulation and depletion are indicated in red and blue, respectively, at an isosurface level of 0.0025 e/Bohr^3^.

Next, we studied the adsorption properties of Configuration II as a function of the molecular coverage ranging from 6% to 50% using 3 × 3 and 4 × 4 supercells. As shown in Figure 3, the total charge transfer per 1 × 1 surface unit cell increases with coverage, primarily due to the cumulative electron uptake by the adsorbed NO_2_ molecules. However, both the average charge transfer and adsorption energy per molecule gradually decrease as the coverage increases, which can be attributed to intermolecular repulsion and steric hindrance. Despite this, the adsorption energy remains greater than 0.6 eV per molecule and the charge transfer is close to 0.7 electron per molecule even after all Se vacancy sites are saturated, indicating a robust and stable adsorption system accompanied by a progressive p-doping effect. The coverage-dependent DOS and the downward shift of the Fermi level in the adsorption system are presented in Figure S4 in the Supporting Information.

**Figure 3:**
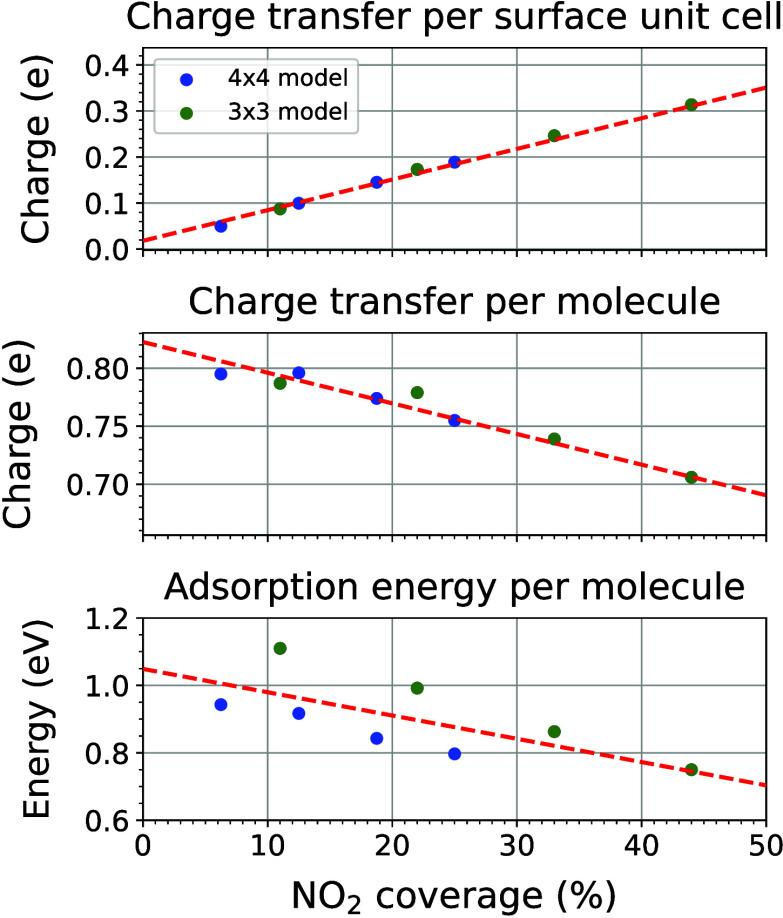
Charge transfer and adsorption energy for NO_2_ adsorption at various surface coverages on the pristine Bi_2_O_2_Se film. Red dashed lines serve as visual guides to highlight the overall trends. The spread in data reflects variations arising from different intermolecular interactions in the 3 × 3 and 4 × 4 surface models. Charge transfer values are obtained from Bader charge analysis..

Given that CVD-grown films usually have Se vacancies, we further studied the adsorption behavior on a defective Bi_2_O_2_Se film with 4.1% V_Se_ and confirmed that the adsorption-induced p-doping effect prevails. The adsorption configurations, including bond lengths and angles, remain largely unchanged compared with those in the pristine system. Bader charge analysis shows that the amount of charge transferred to NO_2_ is also similar in both cases. However, the NO_2_ adsorption energy on the defective film is approximately twice that on the pristine system, indicating an even more stable system. Figure 4(a) shows the projected DOS for the adsorption of 6% and 19% NO_2_ on a defective Bi_2_O_2_Se film with 4.1% V_Se_. Although the overall DOS profile remains the same as that of the pristine film (see Figure 1), the Fermi level moves up to within the conduction band, reflecting the n-type degenerate characteristics induced by internal selenium vacancies, as observed in the experiment.^12,13^ Upon NO_2_ adsorption, the molecule-related states (red lines) are broadened and slightly shifted relative to their isolated molecular levels, indicating hybridization with the Bi_2_O_2_Se electronic states. However, the overall DOS profile for the semiconductor film is not changed and the chemical potential progressively shifts downward, indicating p-type doping driven by electron transfer to the molecule. Given the low DOS at the edge of the conduction bands, the Fermi level shift is noticeable. At 19% coverage, the Fermi level approaches the conduction band minimum (CBM), suggesting a substantial reduction in the carrier concentration.

**Figure 4:**
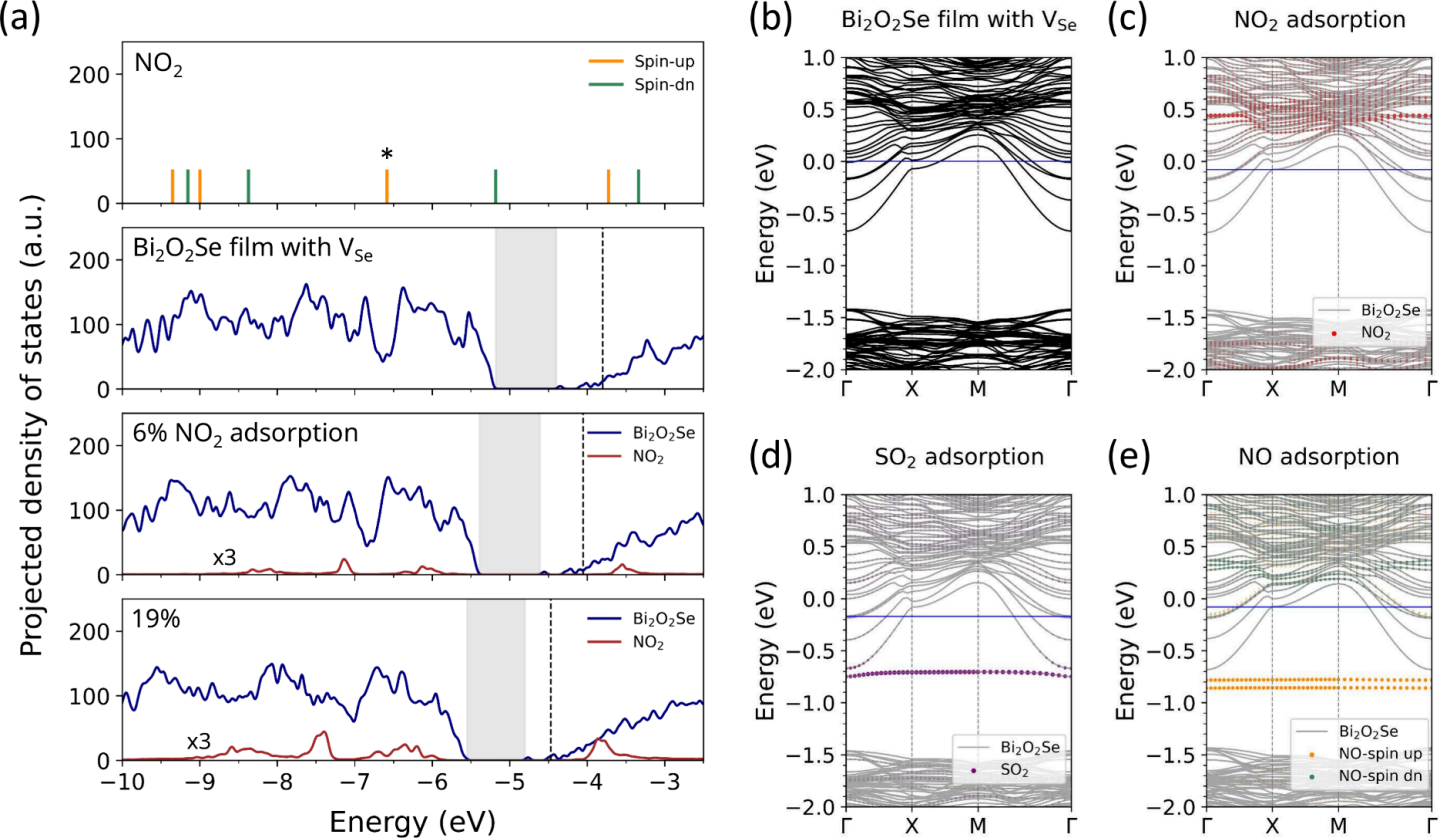
(a) Projected density of states (DOS) for 6% and 19% NO_2_ adsorption on a defective Bi_2_O_2_Se film containing 4.1% V_Se_. For comparison, the DOS of an isolated NO_2_ molecule and the defective film with 4.1% internal Se vacancies are shown above. The HOMO of NO_2_ is marked with an asterisk (*). Black dashed lines indicate the Fermi level in each system, and the band gap regions are shaded in gray. All energies are referenced to the vacuum level, and the surface dipole created by the charge transfer causes an energy shift in the adsorption system. Projected band structures are shown for (b) a defective Bi_2_O_2_Se film with 4.1% V_Se_ and the consequent adsorption systems with a 6% molecular coverage of (c) NO_2_, (d) SO_2_, and (e) NO, with blue lines denoting the Fermi level. Band energies are aligned by deep O 2*s* core levels with energy zero set at the Fermi level of the defective Bi_2_O_2_Se film as in (b).

Figure 4(b) shows the band structure of the defective Bi_2_O_2_Se film with 4.1% V_Se_ and Figure 4(c) of the adsorption system with 6% NO_2_ calculated using a 4 × 4 supercell. The adsorption does not change the overall band dispersions but only induces a shift of the Fermi level. Other NO_2_-induced states are located approximately 0.5 eV above the CBM, well beyond the active transport regime, thus minimizing the risk of transport interference and performance degradation. A careful examination of the layer-by-layer changes in charge density after adsorption reveals that although significant charge transfer occurs between NO_2_ and neighboring Bi atoms on the surface, charge depletion actually extends to a few chemical layers below. For a 6% coverage of NO_2_ on the defective Bi_2_O_2_Se film with 4.1% V_Se_, the calculated total charge transfer is 0.8 electron, and approximately 0.44 among them is from the top layers of Se and Bi. The remaining 0.36 electron is drawn mainly from deeper Bi layers, consistent with the orbital features near the CBM.

This finding indicates that in addition to the local interaction between the molecule and the surface, adsorption of NO_2_ can effectively lead to p-type doping in the first few layers of the semiconductor, namely, the channel layers in a thin film device. It is consistent with the previous experimental observation that resistivity increases upon NO_2_ exposure in Bi_2_O_2_Se thin films.^22^ This adsorption-induced p-type doping offers a unique and effective strategy to lower the threshold voltage and modulate the switching behavior in Bi_2_O_2_Se-based electronic devices, as will be experimentally demonstrated later in this work. NO_2_ is a particularly good candidate due to the favorable alignment of the energy levels, its positive electron affinity, and its high adsorption energy.

Among other molecules, SO_2_ and NO have the LUMO within the band gap of Bi_2_O_2_Se, as shown in Figure 1. Hence, charge transfer from the internal semiconductor layers is not expected upon adsorption on the pristine film. Although our calculations found charge transfers of approximately 0.51 and 0.24 electron per molecule for SO_2_ and NO, respectively, almost all are from the surface layers with no contribution from the internal layers and no effect expected on the device performance. The situation changes when SO_2_ or NO adsorption occurs on the defective Bi_2_O_2_Se film with 4.1% V_Se_, where the original Fermi level is in the conduction band and above the molecular LUMO. In addition to charge transfer due to localized surface interaction, charge depletion was also found to extend to a few chemical layers below, just as in the case of NO_2_ adsorption. This results in a downward shift of the Fermi level, as shown in the band structure in Figure 4(d) and 4(e). The overall band dispersions remain largely intact, but localized molecular states can be found below the CBM in the gap for both SO_2_ and NO. The adsorption configurations and electronic DOS are presented in Figures S5 and S6, respectively, in the Supporting Information.

The LUMO of Cl_2_ is deep within the valence band of Bi_2_O_2_Se (see Figure 1), and in our calculation the molecule dissociates after adsorption on the surface. (Details are provided in Figure S9 in the Supporting Information.) NF_3_ was also found to disintegrate on the surface of the Bi_2_O_2_Se film, with internal Se vacancies. Among the remaining molecules studied, NH_3_, CO_2_, and N_2_ all exhibit physisorption behavior on the Bi_2_O_2_Se surface, characterized by a small adsorption energy and minimal charge transfer on both pristine and defective (with internal Se vacancies) films. As an example, the adsorption geometry and the difference charge density plot for NH_3_ is shown in Figure 2(d). Although the calculated adsorption energy is 0.43 eV, charge transfer is minimal, as confirmed by Bader charge analysis. Charge redistribution is largely confined within the NH_3_ molecule, and negligible charge transfer is also reflected in the unshifted Fermi level in the DOS for the case of 11% NH_3_ adsorption (see Figure S7 in the Supporting Information).

Our experimental findings described in the following show that the adsorption of NO_2_ on the surface indeed induces a favorable p-doping effect and that the adsorption of NH_3_ does not. We adopted a threshold-voltage (V_th_) modulation FET architecture that employs CVD-grown Bi_2_O_2_Se as the active channel material, with NO_2_ serving as a controllable surface dopant. As shown in Figure 5(a), Raman spectroscopy confirmed the crystalline quality of the as-grown film through a sharp A_1*g*_ mode at 159 cm^–1^, while the 521 cm^–1^ peak corresponds to the SiO_2_/Si substrate. The high-angle annular dark-field (STEM-HAADF) image of cross-sectional scanning transmission electron microscopy [Figure 5(b)] and the atomic force microscopy (AFM) data [Figure 5(d)] verified a layered structure with a thickness of about 15.5 nm, equivalent to 25 stacked Bi_2_O_2_Se layers. The optical micrograph of the fabricated FET device [Figure 5(c)] highlights the uniform geometry and well-defined channel region.

**Figure 5:**
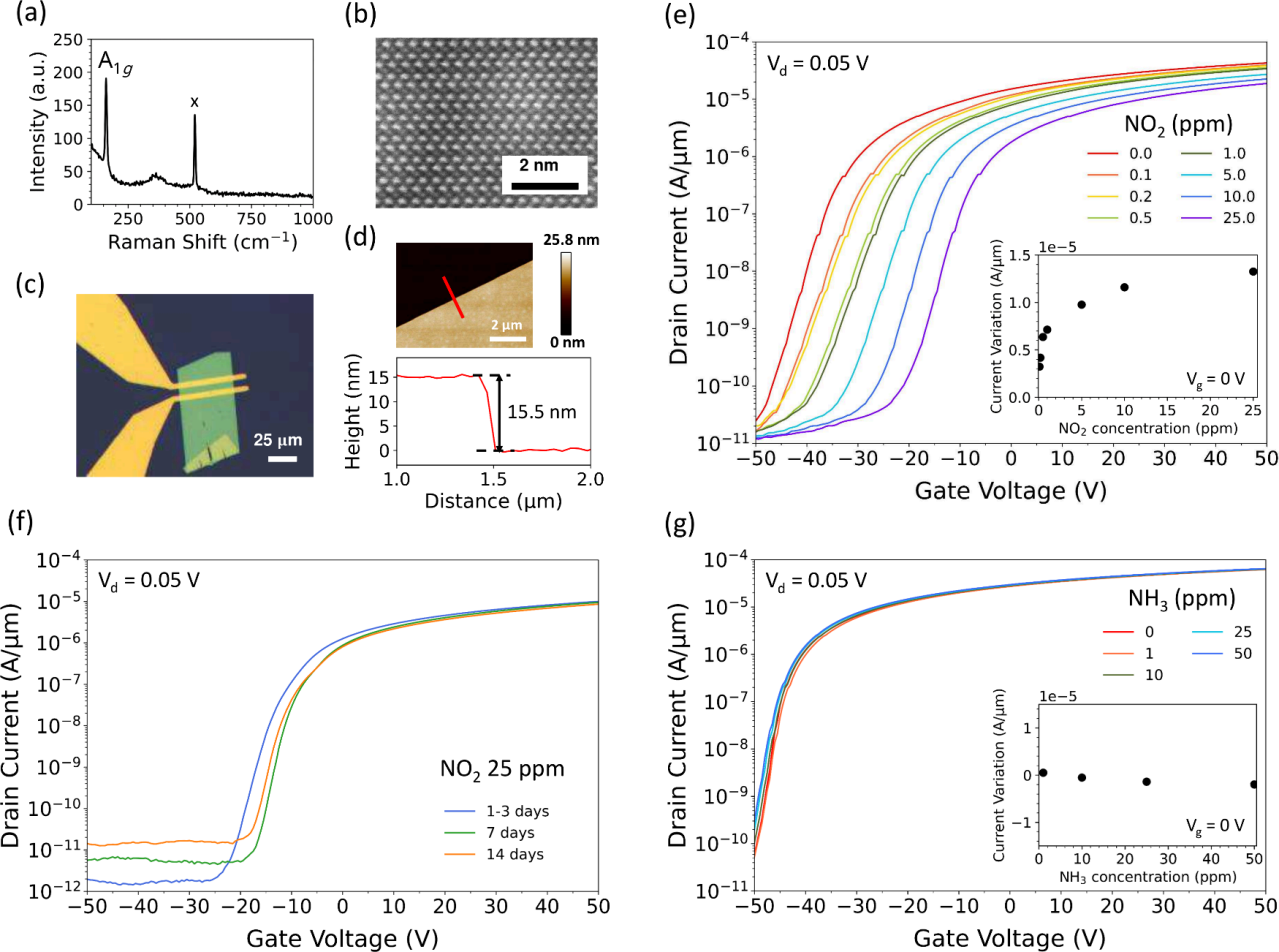
(a) Raman spectrum of the as-grown Bi_2_O_2_Se showing the A_1*g*_ peak at 159 cm^**–**1^. (b) Cross-sectional STEM-HAADF of a Bi_2_O_2_Se nanoplate. (c) Optical image of the fabricated device. (d) Step-height profile measured with the atomic force microscopy (AFM) image, confirming a thickness of 15.5 nm. (e) Transfer characteristics of Bi_2_O_2_Se FETs under varying NO_2_ concentrations, exhibiting a positive threshold voltage shift with increasing NO_2_ partial pressures. The inset shows a logarithmic increase in current variation compared to that in the undoped sample, indicating the decreasing carrier concentration and saturating doping effect with higher NO_2_ exposure. (f) Temporal evolution of the transfer characteristics of the 25 ppm of NO_2_-doped device, demonstrating excellent stability after 14 days. (g) Transfer characteristics of Bi_2_O_2_Se FETs exposed to different NH_**3**_ concentrations, showing negligible threshold voltage shifts. The inset confirms minimal current variation across NH_3_ concentrations. These electrical measurements are carried out under a drain voltage of 0.05 V.

Figure 5(e) presents the transfer characteristics of Bi_2_O_2_Se FETs subjected to varying concentrations of NO_2_ in the adsorption process. Before NO_2_ adsorption, the (undoped) device exhibits an initial V_th_ of approximately −40 V, indicating that it is a highly n-type degenerate semiconductor. With NO_2_ adsorption, the transfer curves progressively shift toward more positive gate voltages, demonstrating a tunable p-type doping effect and enhanced electrostatic control. As illustrated in the inset, the current variation, quantified as $\left|I_{d,w/NO_{2}}-I_{d,w/oNO_{2}}\right|$ at *V*_g_ = 0 V, exhibits a logarithmic increase with respect to the NO_2_ concentration. This trend confirms the features of a reduced electron carrier density and a gradually saturating doping effect at higher NO_2_ exposures.

Most importantly, the NO_2_-induced doping effect exhibits an excellent stability. As shown in Figure 5(f), devices following exposure of 25 ppm of NO_2_ retain their electrical properties with less than a 10 V shift in V_th_ after 14 days under ambient conditions. This persistent doping effect is consistent with the large adsorption energy of the NO_2_ molecules obtained by our DFT calculations. In contrast, devices exposed to NH_3_ show negligible threshold voltage changes [Figure 5(g)]. The inset confirms minimal current variation across NH_3_ concentrations, indicating insignificant carrier modulation.

In summary, our combined theoretical and experimental work has demonstrated that the surface adsorption of molecules can induce substantial and tunable doping effects in the thin-film semiconductor Bi_2_O_2_Se. First-principles calculations reveal that charge transfer may occur not only locally at the surface but also within the underlying semiconductor layers, enabling modulation of transport properties in the channel. Our theoretical and experimental findings collectively establish NO_2_ as a highly effective and sustainable chemical dopant. This adsorption-induced p-doping approach successfully overcomes the key challenges of poor electrostatic control and high threshold voltage in Bi_2_O_2_Se FETs, paving the way for their integration into next-generation nanoelectronic devices.

## Supplementary Material



## Notes

Experimental and computational methods; details of atomic configurations in the calculations; NO_2_ adsorption on pristine Bi_2_O_2_Se; adsorption energies and charge transfer analysis of various molecules (PDF)
