# Supplementary material for: Sustainable Doping via Molecular Adsorption on Thin-Film Semiconductor Bi2O2Se
Source: Nano Lett. 2025 Dec 3;25(50):17341–7. doi: 10.1021/acs.nanolett.5c04613 (PMC12715839; doi:10.1021/acs.nanolett.5c04613)
Supplement: Supplementary file 1 [file nl5c04613_si_001.pdf]

Supporting Information

Sustainable doping via molecular adsorption on thin-film semiconductor Bi_2O_2Se

Tai-Ting Lee,[†,‡] Chi-Chun Cheng,[¶] Tzu-Wen Kuo,[§] Shu-Yu Hsu,[‖] Chun-Yen Hsiao,[‖] Yu-Lun Chueh,[§] Po-Wen Chiu,[*,‡,¶] and Mei-Yin Chou[*,†,‡]

†*Department of Physics, National Taiwan University, Taipei 10617, Taiwan*

‡*Institute of Atomic and Molecular Sciences, Academia Sinica, Taipei 10617, Taiwan*

¶*Department of Electrical Engineering, National Tsing Hua University, Hsinchu 30013, Taiwan*

§*Department of Materials Science and Engineering, National Tsing Hua University, Hsinchu 30013, Taiwan*

‖*College of Semiconductor Research, National Tsing Hua University, Hsinchu 30013, Taiwan*

E-mail: pwchiu@ee.nthu.edu.tw; mychou6@gate.sinica.edu.tw

Methods

Chemical Vapor Deposition (CVD) Synthesis of Bi_2O_2Se Films

Single-crystalline Bi_2O_2Se thin films were synthesized via low-pressure chemical vapor deposition (LPCVD). High-purity Bi_2O_3 powder and Bi_2Se_3 bulk crystals (5N, both from Alfa Aesar) were used as precursors and placed in a chessboard pattern at the center of a quartz boat in the hot zone of the furnace. To remove residual moisture, the precursors were preheated to 120 ℃ for 20 minutes before the growth process. During growth, 200 sccm of argon was introduced as the carrier gas to transport the vapor-phase precursors downstream to the deposition zone, where a fluorophlogopite mica (f-mica) substrate was placed. The growth was conducted at 580 ℃ under a system pressure of 400 torr for a duration of 8 minutes. The growth time was found to be a critical parameter for controlling film thickness; in particular, an 8-minute duration yielded Bi_2O_2Se films with thicknesses ranging from 10 nm to 100 nm.

Characterization of Bi_2O_2Se Films

To evaluate the structural and morphological properties of the Bi_2O_2Se films, several characterization techniques were employed. Raman spectroscopy was performed using a Thermo Scientific DXR Raman Microscope with a 523 nm excitation laser at a power of 10 mW to confirm the crystallinity of the as-grown films. Atomic resolution cross-sectional imaging was conducted by scanning transmission electron microscopy in the high-angle annular dark-field mode (STEM-HAADF) using an FEI Talos F200X operated at 300 kV. The film thickness was measured using a scanning probe microscope (Bruker Dimension ICON) with a scanning rate of 0.999 Hz. The system maintained an XY noise level below 0.15 nm and a Z sensor noise level below 0.35 Å, ensuring high-resolution topographic accuracy.

Device Fabrication and Measurements

To ensure semiconducting behavior, Bi_2O_2Se crystals with thicknesses ranging from 10 to 30 nm were selected for channel materials. Free-standing Bi_2O_2Se nanoplates, synthesized via CVD on mica substrates, were mechanically transferred onto a 90 nm SiO_2/P^+Si substrate. Back-gated FETs were fabricated using standard photolithography techniques. A bilayer photoresist (LOR/S1813) was spin coated to define the source and drain regions, followed by e-beam evaporation of a Ti/Au (10/40 nm) metal stack at a deposition rate of 1 Å/s under a base pressure of 3×10^{-6} Torr.

After fabrication, the devices were placed in a vacuum probe station maintained at 5×10^{-2} Torr for gas exposure and electrical characterization. The target gas species were introduced into the chamber at a flow rate of 1000 sccm, and the system pressure was stabilized at 500 Torr. Following equilibration, doping was carried out by exposing the devices to the gas for 10 minutes at each concentration level prior to electrical measurements. Notably, for the evolution of the transfer curves shown in Figure 5(f), the device was initially exposed to 25 ppm NO_2 for 90 minutes to strengthen the doping effect. All electrical measurements were performed at room temperature using a semiconductor analyzer (Keysight B1500A).

First-principles calculation

We performed first-principles calculations within the framework of density functional theory (DFT) to investigate the structural and electronic properties of molecular adsorption on the Bi_2O_2Se surface. All DFT simulations were carried out using the Vienna Ab initio Simulation Package (VASP),[1] employing the exchange-correlation functional proposed by Perdew, Burke, and Ernzerhof (PBE).[2] The effects of core electrons were described using the projector-augmented wave (PAW) method,[3] with a plane-wave energy cutoff set to 600 eV. Data post-processing was conducted using VASPKIT.[4]

A 3×3 or 4×4 slab with a thickness of three Bi_2O_2Se chemical layers was used to explore various adsorption configurations. Adding an additional layer only changes the adsorption

energy by less than 0.05 eV per molecule. The model with 4.1% internal Se vacancies was constructed by removing two Se atoms from the inner layers. All slab models included a vacuum layer exceeding 18 Å to eliminate spurious interactions between periodic images. Structural relaxation was performed until the atomic force on each atom was less than 0.01 eV/Å, and the total energy was converged to 1×10^{-5} eV per supercell. For self-consistent calculations, Monkhorst-Pack k-point grids of $3\times3\times1$ and $2\times2\times1$ were used for the 3×3 and 4×4 supercells, respectively, corresponding to a k-spacing of 0.03 (2π/Å) in each case.

A previous characterization of the cleaved surface by scanning tunneling microscopy (STM) concluded that the film terminates in the Se plane with 50% Se coverage and 50% Se-vacancy dimers on both sides.[5] Therefore, we considered surface models containing Se vacancy dimers accordingly. To determine an energetically optimal arrangement for the 3×3 and 4×4 supercells, we calculated the cleavage surface energies of various configurations illustrated in Figure S1.

For the 3×3 supercell, panels (a)–(d) show four different vacancy arrangements on one surface with complementary arrangements on the other to ensure a total of 100% Se coverage on two surfaces with charge neutrality. The average surface energy per unit cell and the Se coverage on each surface are shown. Similarly, for the 4×4 supercell, panels (e)–(h) show the possible configurations with relevant information. Among the configurations explored in Figure S1, (a) and (e) exhibit the lowest surface energies, consistent with the vacancy dimer characteristics observed in STM. Therefore, they are selected for the adsorption surface in our calculations.

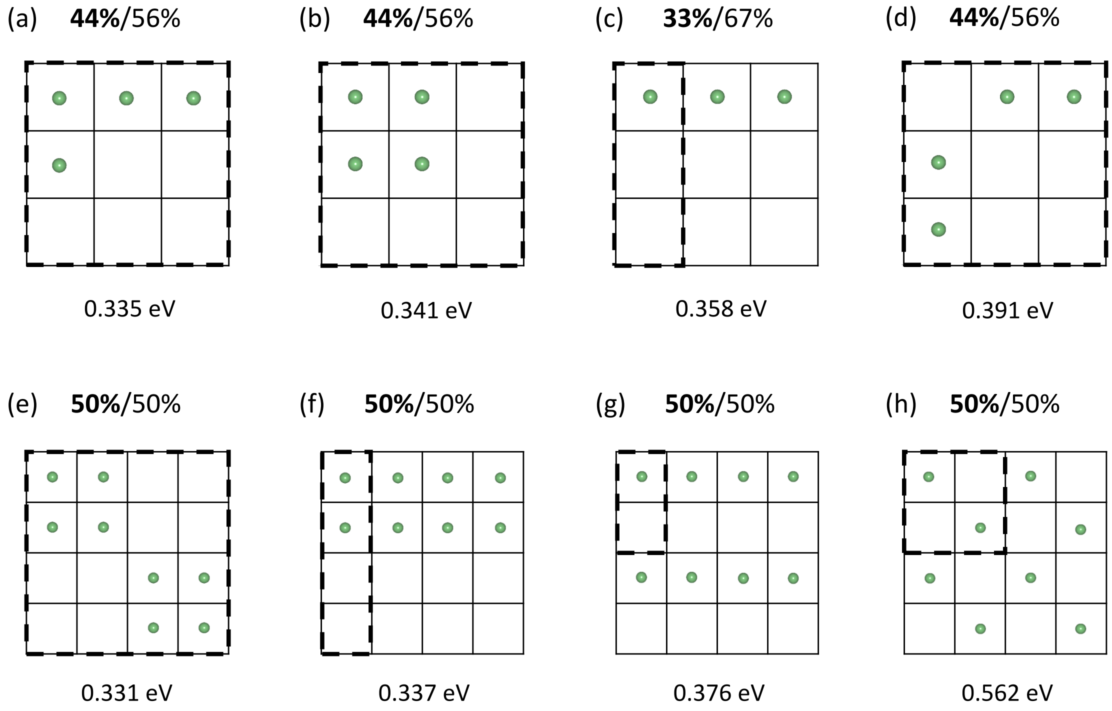

Figure S1: **Various Se arrangements of the slab surfaces: (a)–(d) in the 3×3 supercell and (e)–(h) in the 4×4 supercell. The small squares represent possible Se sites, and the occupied ones are labeled with green dots on one surface, with the arrangement on the opposite surface reversed (not shown). The Se coverages on the two surfaces are shown, with a sum of 100% to ensure charge neutrality. The black dashed lines indicate the calculational unit cell, and the calculated cleavage energy per 1×1 unit cell is also shown.**

Additional Adsorption Properties

Different adsorption sites

Figure S2 compared the calculated adsorption energies of 11% NO_2, SO_2 and NH_3 at different surface sites: at a Se vacancy, above a Se site, and above a Bi site. It can be seen that for all three molecules the highest adsorption energy occurs at a Se vacancy site, because the extra space allows the molecule to develop a tighter binding with the surface.

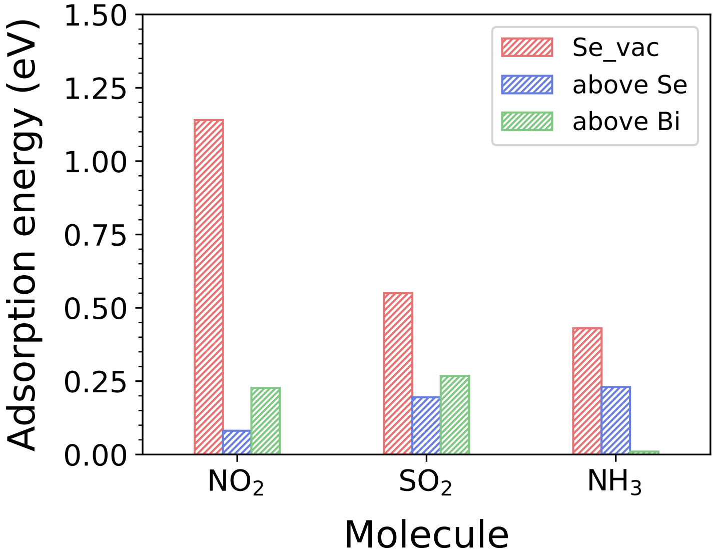

Figure S2: **Adsorption energy of NO_2, SO_2, and NH_3 at different adsorption sites (at a Se vacancy, above a Se site, and above a Bi site) on the Bi_2O_2Se surface in the 3×3 supercell (11% coverage).**

For the surface model in Figure S1(a), there are different Se vacancy sites on the surface with different local environments. We calculated the adsorption energy of NO_2 in Configuration II [Figure 2(c)] at three different Se vacancy sites as shown in Figure S3. The results for charge transfer and adsorption energy are all similar.

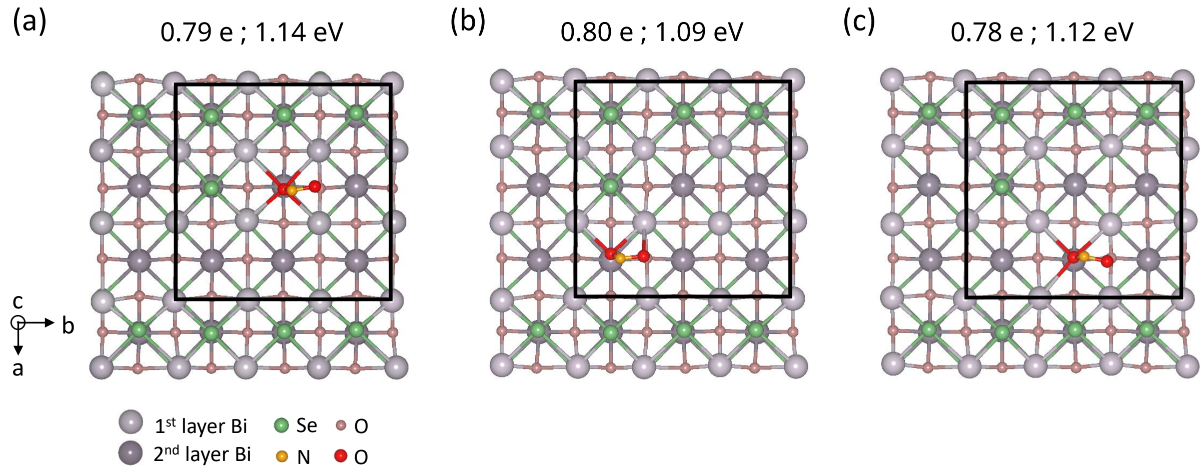

Figure S3: **Local atomic configurations of NO_2 at three representative adsorption sites with different environments on the Bi_2O_2Se surface: the Se-vacancy sites in (a) and (b) have two adjacent Se atoms, while that in (c) has one. The black outline marks the boundary of the 3×3 supercell with a surface Se arrangement as in Figure S1(a). The charge transfer and adsorption values are marked.**

NO_2 adsorption on the pristine Bi_2O_2Se film

Figure S4 shows the projected density of states (PDOS) of NO_2 adsorption systems with coverages from 11% to 33% on the pristine Bi_2O_2Se film (no internal Se vacancies). For comparison, the spin-polarized DOS of an isolated NO_2 molecule and that of pristine Bi_2O_2Se are also plotted, where the molecular HOMO is marked with an asterisk (*). The energy zero is set at the vacuum level, and the surface dipole created by the charge transfer upon adsorption causes an energy shift in the adsorption systems. The gray-shaded region indicates the band gap, and the Fermi level is denoted by a dashed line in each system. It can be seen that the free NO_2 molecule has its LUMO below the band gap of Bi_2O_2Se, thereby it is expected to gain electrons upon adsorption.

The interaction between NO_2 and Bi_2O_2Se gives rise to a broadening and a shift of the molecular states in the DOS plot. The calculated total charge transfer based on Bader charge analysis for the 11% coverage is 0.79 electrons and approximately 0.39 are from the top layers of Se and Bi. The remaining 0.40 electrons are drawn mainly from deeper Se layers, consistent with the orbital features near the valence band maximum (VBM). Therefore, progressively downward shifts of the Fermi level in the film can be seen as the NO_2 coverage increases in Figure S4.

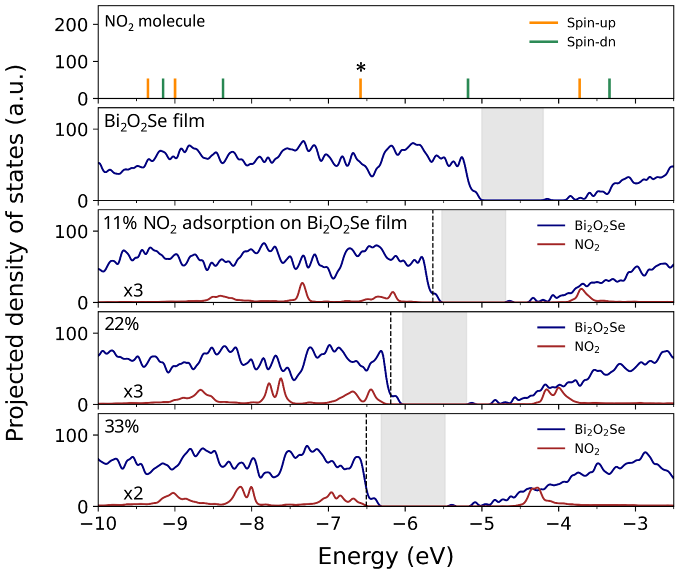

Figure S4: **Projected density of states of NO_2 adsorption systems with coverages from 11% to 33% on the pristine Bi_2O_2Se film (no internal Se vacancies). The density of states of an isolated NO_2 molecule and that of the pristine film are also shown for comparison. The HOMO of NO_2 is marked with an asterisk (*). Black dashed lines indicate the Fermi level in each system, and the band gap regions are shaded in gray. All energies are referenced to the vacuum level.**

Adsorption of SO_2 and NO

Similar to NO_2, SO_2 is also a polar molecule; however, only one stable adsorption configuration is found on the pristine Bi_2O_2Se film with no internal vacancies, with an adsorption energy of 0.71 eV per molecule [Figure S5(a)]. The length between O in SO_2 and Bi in the top layer is 2.79–2.86 Å, where clear charge accumulation and depletion can be seen, resulting in a charge transfer of 0.51 electrons per molecule mainly from localized surface interactions with little changes in the inner semiconductor layers. The molecular orientation is similar when adsorption occurs on the Bi_2O_2Se film with 4.1% V_{Se}, while the bonding between SO_2 and the surface becomes stronger, showing O-Bi bonds of 2.52–2.63 Å with a large adsorption energy of 1.57 eV per molecule and a total charge transfer of 1.23 electrons per molecule.

NO undergoes a weaker adsorption, floating slightly above the pristine Bi_2O_2Se film with a small adsorption energy of 0.2 eV per molecule and a charge transfer of 0.2 electrons per molecule [Figure S5(b)]. For adsorption on the Bi_2O_2Se film with 4.1% V_{Se}, where the Fermi level lies above the LUMO of a NO molecule, it changes to chemisorption with a considerable adsorption energy of 1.39 eV. This leads to shorter N-Bi bonds of 2.87–3.02 Å [Figure S5(c)], and a charge gain of 0.62 electrons per molecule.

Figure S6 shows the PDOS for 6% and 19% SO_2 and NO adsorption on the Bi_2O_2Se film with 4.1% V_{Se}. For comparison, the DOS of each isolated molecule and the film with 4.1% V_{Se} are shown for comparison. The highest occupied molecular orbital (HOMO) is marked with an asterisk (*), and the partially occupied state is indicated by a half-filled circle (◐). The black dashed line indicates the Fermi level in each system, and the band-gap regions are shaded in gray. As in the case of NO_2, SO_2 and NO also draw electrons from the internal layers of the Bi_2O_2Se film with 4.1% V_{Se} based on Bader charge analysis, leading to a progressive downward shift in the Fermi level of the adsorption systems as shown in Figure S6. However, the localized molecular states in the gap may have undesirable drawbacks.

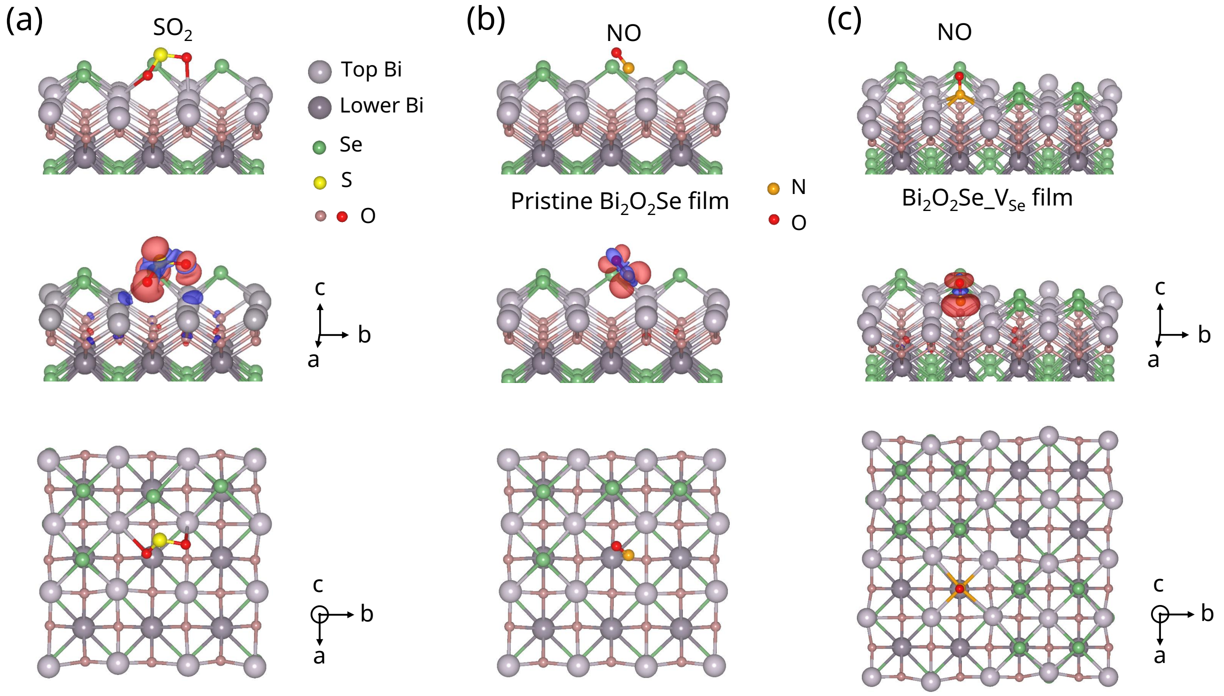

Figure S5: **(a) Optimal structure of SO_2 adsorption on the pristine Bi_2O_2Se film (no internal Se vacancies); NO adsorption on (b) the pristine Bi_2O_2Se film and (c) the Bi_2O_2Se film with 4.1% V_{Se} (side and top views), with the corresponding difference charge density plot at an isosurface level of 0.0025 e/Bohr^3.**

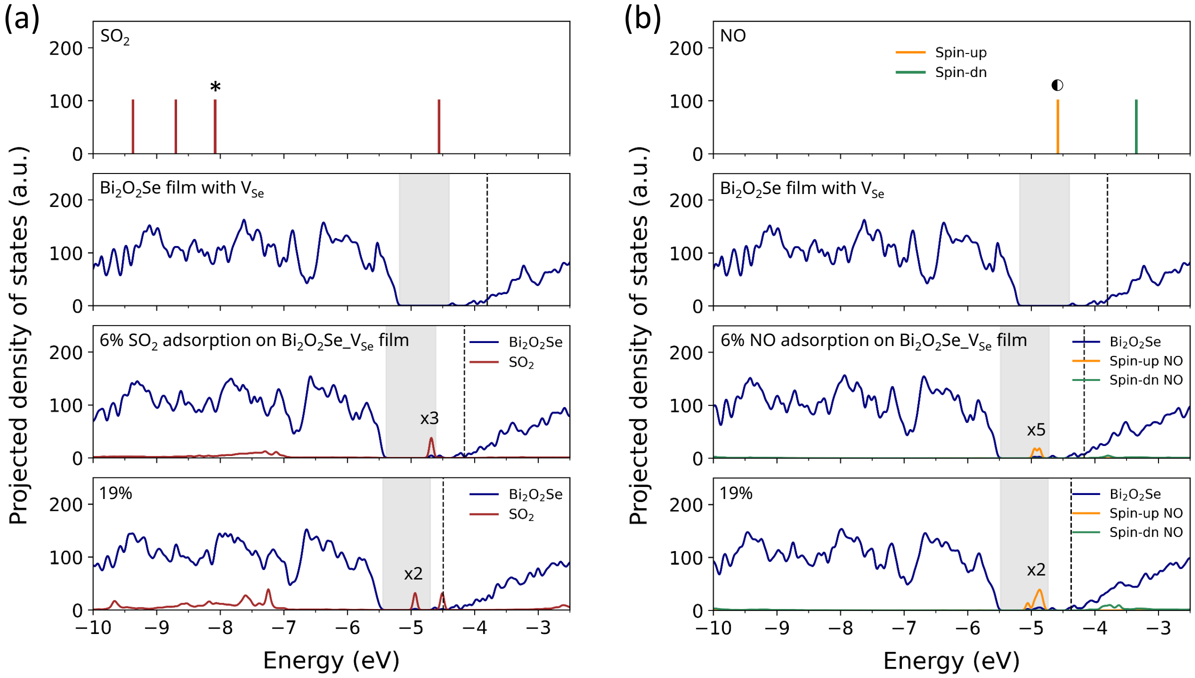

Figure S6: **Projected density of states of the adsorption systems with 6% and 19% (a) SO_2 and (b) NO on the Bi_2O_2Se film with 4.1% V_{Se}. For comparison, the densities of states of isolated molecules and the defective film are also shown. The highest occupied molecular orbital (HOMO) is marked with an asterisk (*), and a partially occupied state is indicated by a half-filled circle (◐). The black dashed line indicates the Fermi level in each system and the band-gap regions are shaded in gray. All energies are referenced to the vacuum level.**

Adsorption of NH_3

Figure S7 shows the PDOS of the adsorption system with 11% NH_3 on the surface of the pristine Bi_2O_2Se film (no internal vacancies). It can be seen that the Fermi level of the adsorption system remains intact compared to that of the pristine film, and the HOMO state of NH_3 is also nearly intact after adsorption. This can be attributed to the fact that the LUMO of NH_3 is too high (outside the energy range in Figure S7) to facilitate any chemical interaction and charge transfer.

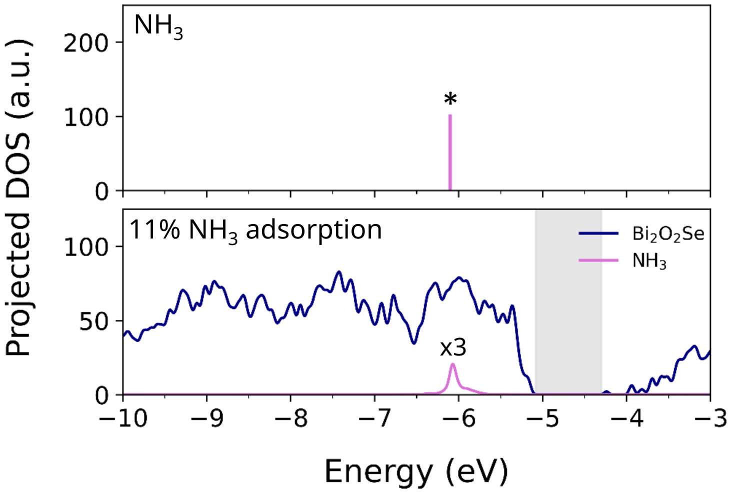

Figure S7: **Projected density of states of the adsorption system with 11% NH_3 on the pristine Bi_2O_2Se film. The density of states of an isolated NH_3 molecule is also shown for comparison. The asterisk (*) denotes the HOMO of NH_3. The gray-shaded region indicates the band gap of Bi_2O_2Se. All energy levels are referenced to the vacuum level.**

Adsorption energy and charge transfer

Figure S8 summarized the adsorption energy and charge transfer for molecules listed in Figure S8. Cl_2 and NF_3 are not included due to the fact that they are decomposed on the Bi_2O_2Se film. NH_3, CO_2, and N_2 show small adsorption energy and charge transfer on both the pristine film and the defective film with 4.1% V_{Se}.

A significant increase in the adsorption energy is found for the adsorption of NO_2, SO_2, and NO on the defective Bi_2O_2Se film with 4.1% V_{Se} compared to the adsorption on the pristine film (no internal Se vacancies) [Figure S9(a)]. For NO_2, this increase is attributed to the different trends in the DOS near the VBM and the CBM of Bi_2O_2Se , which leads to a smaller Fermi energy shift near the VBM for NO_2 adsorption on the pristine film and a larger shift near the CBM for adsorption on the defective film with similar charge transfer results as shown in Figure S8(b). As a result, the total energy change upon adsorption on the defective film is greater than that on the pristine film.

In contrast, the situation is different for SO_2 and NO. These molecules only draw charges mainly from the top chemical layer upon adsorption on the pristine Bi_2O_2Se film, as indicated by Bader charge analysis. This local interaction does not modify the Fermi level of the adsorption system. On the other hand, the Fermi level of the film with 4.1% V_{Se} is above the LUMO of SO_2 and NO as shown in Fgure S6. Therefore, the resulting charge transfer and adsorption energy of these adsorption systems increase significantly to more than twice that found on the pristine film.

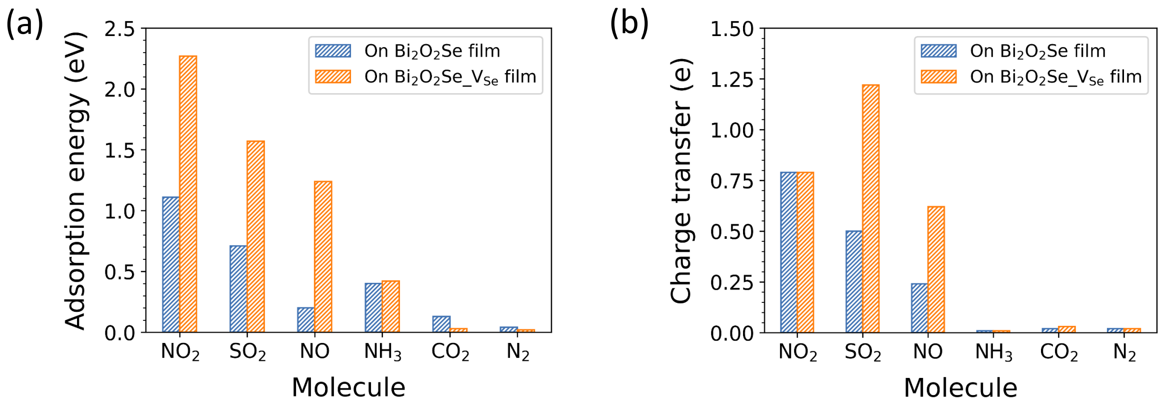

Figure S8: **(a) Adsorption energy and (b) charge transfer for different molecules adsorbed on the Bi_2O_2Se films without internal Se vacancies and with 4.1% V_{Se}. The results are for a coverage of 6%.**

Adsorption of Cl atom

In our calculations, it is found that the Cl_2 molecule will dissociate upon adsorption on the Bi_2O_2Se surface to reach the stable configuration involving individual Cl atoms on the surface Se vacancy sites [Figure S9(a)]. The adsorption energy is 0.97 eV per atom with respect to the molecular state. The distance between the Cl atom and the top-layer Bi atoms is 3.12–3.15 Å, where clear charge accumulation and depletion can be seen in Figure S9(a).

Figure S9(b) shows the band structure of the defective Bi_2O_2Se film with 4.1% V_{Se} as a reference, while Figure S9(c) shows that of the adsorption system with 6% Cl atom coverage calculated using a 4×4 supercell. The adsorption does not alter overall band dispersions nor induce localized states within the gap, but only creates a shift of the Fermi level. Bader charge analysis reveals a total charge transfer of 0.70 electrons, with 0.34 electrons originating from the top Bi and Se layers and the remaining 0.36 electrons drawn from the inner layers. Therefore, Cl atom adsorption can also induce a p-doping effect in Bi_2O_2Se, similar to the case of NO_2.

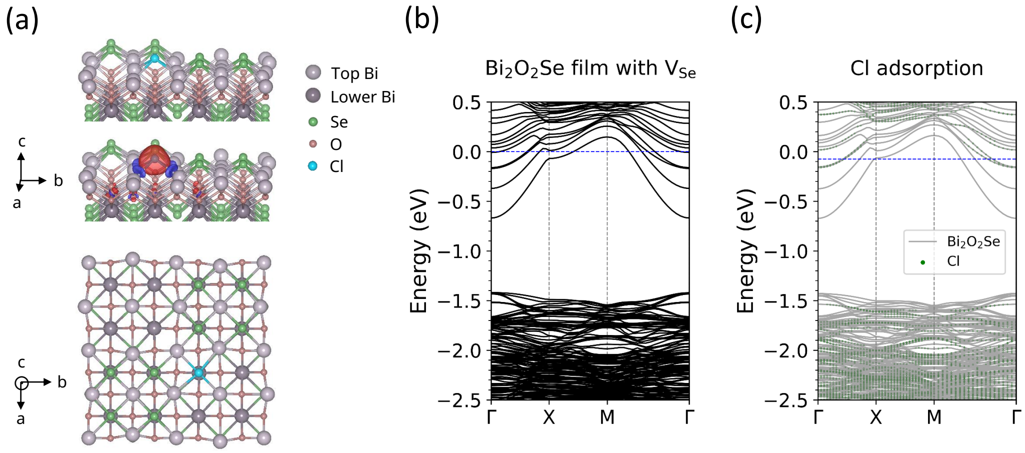

Figure S9: **(a) Optimal structure of single Cl atom adsorption on the Bi_2O_2Se film with 4.1% V_{Se} (side and top views), with the corresponding difference charge density plot at an isosurface level of 0.0025 e/Bohr^3. (b) Band structure of a defective Bi_2O_2Se film with 4.1% V_{Se} and (c) Projected band structures for 6% Cl atom adsorption, with blue lines denoting the Fermi level. Band energies are aligned by deep O 2*s* core levels with energy zero set at the Fermi level of the defective Bi_2O_2Se film as in (b).**

References

(1) Kresse, G.; Furthmüller, J. Efficient Iterative Schemes for *Ab initio* Total-Energy Calculations Using a Plane-Wave Basis Set. *Phys. Rev. B* **1996**, *54*, 11169.

(2) Perdew, J. P.; Burke, K.; Ernzerhof, M. Generalized Gradient Approximation Made Simple. *Phys. Rev. Lett.* **1996**, *77*, 3865.

(3) Blöchl, P. E. Projector Augmented-Wave Method. *Phys. Rev. B* **1994**, *50*, 17953.

(4) Wang, V.; Xu, N.; Liu, J.-C.; Tang, G.; Geng, W.-T. VASPKIT: A User-Friendly Interface Facilitating High-Throughput Computing and Analysis Using VASP Code. *Comput. Phys. Commun.* **2021**, *267*, 108033.

(5) Chen, C.; Wang, M.; Wu, J.; Fu, H.; Yang, H.; Tian, Z.; Tu, T.; Peng, H.; Sun, Y.; Xu, X.; Jiang, J.; Schröter, N. B. M.; Li, Y.; Pei, D.; Liu, S.; Ekahana1, S. A.; Yuan, H.; Xue, J.; Li, G.; Jia, J.; Liu, Z.; Yan, B.; Peng, H.; Chen, Y. Electronic Structures and Unusually Robust Bandgap in An Ultrahigh-Mobility Layered oxide Semiconductor, Bi_2O_2Se. *Sci. Adv.* **2018**, *4*, eaat8355.
